# Interlayer Adhesion Analysis of 3D-Printed Continuous Carbon Fibre-Reinforced Composites

**DOI:** 10.3390/polym13101653

**Published:** 2021-05-19

**Authors:** Tomas Kuncius, Marius Rimašauskas, Rūta Rimašauskienė

**Affiliations:** Faculty of Mechanical Engineering and Design, Kaunas University of Technology, Studentų Str. 56, 51424 Kaunas, Lithuania; tomas.kuncius@ktu.lt (T.K.); ruta.rimasauskiene@ktu.lt (R.R.)

**Keywords:** interlayer adhesion, 3D printing, composites, continuous carbon fibre

## Abstract

Carbon fibre-reinforced materials are becoming more and more popular in various fields of industries because of their lightweight and perfect mechanical properties. Additive manufacturing technologies can be used for the production of complex parts from various materials including composites. Fused deposition modelling (FDM) is an excellent technology for the production of composite structures reinforced with short or continuous carbon fibre. In this study, modified FDM technology was used for the production of composites reinforced with continuous carbon fibre. The main aim of this study is to evaluate the shear strength of 3D-printed composite structures. The influence of printing layer height and line width on shear strength was analysed. Results showed that layer height has a significant influence on shear strength, while the influence of printing line width on shear strength is slightly smaller. Reduction of layer height from 0.4 mm to 0.3 mm allows increasing shear strength by about 40 percent. Moreover, the influence of the shear area and overlap length on shear force showed linear dependency, in which the shear area is increasing the shear force increasing proportionally. Finally, the results obtained can be used for the design and development of new 3D-printed composite structures.

## 1. Introduction

Additive manufacturing (AM) is one of the fastest-growing nontraditional manufacturing technologies. Emerged in the 1980s and limited to prototyping, AM is now increasingly used in many industries such as medicine, aviation, or automotive industries [1,2,3]. All AM technologies allow the creation of 3D objects layer by layer and eliminate the waste generated during production almost completely. On the other hand, the unlimited freedom in geometrical shapes and complexity of printed objects allows these technologies to compete with reasonably, or even overtake, traditional ones [4]. For a long time, the development and wider use of AM technologies have been limited by the poor selection of printing materials, which were mostly polymers; however, currently, it can be used for a very wide range of materials from thermoplastics to metals [5,6], ceramics [7,8,9], and biocompatible [10] or composite materials [11,12]. Composite materials, especially reinforced materials using continuous carbon fibre, have been recently introduced to additive manufacturing. According to ISO/ASTM 52900 (2017) (Additive manufacturing—General principles—Terminology), fused deposition modelling (FDM) is classified as one of the material extrusion-based processes. Moreover, FDM is one of the most often-used AM technologies. Usually, polymer materials such as polylactic acid (PLA) [13,14], acrylonitrile butadiene styrene (ABS) [15], and polycarbonate (PC) [16] are used with this technology. Constantly increasing requirements for produced parts in every field of industry ask for better mechanical properties and lower mass of the parts. These requirements can be met by using relatively new materials such as polyetheretherketone (PEEK) [17,18] and high-density polyethylene (HDPE) [19,20], or by improving the properties of existing materials [21]. For the improvement of mechanical properties, thermal treatment can be used; however, only a slight increase of maximum bending force was observed for PLA samples [13], while the tensile strength increased from 24.7 to 57.3 MPa for polyphenylene sulphide (PPS) material [22]. Though such an increase of mechanical properties is still too small, especially for structural parts, it opens a huge potential niche for composite materials.

Due to its technological features, such as the possibility to use various materials and easy process control, FDM is an excellent technology for the production of composite structures reinforced using continuous carbon fibre [23]. The mechanical properties of printed products can be increased several times by embedding carbon fibre tow into a thermoplastic matrix (PLA, ABS, PC, etc.). For example, flexural strength and tensile strength of PLA samples printed using FDM technology are 53 MPa and 28 MPa, respectively [24], while with inserted continuous carbon fibre tow into the structure the flexural strength can be increased to 156 MPa and tensile strength to 91 MPa [25]. It worth mentioning that modified carbon fibre tow was used during this investigation. Moreover, having inserted only 6.6% of carbon fibre, the tensile strength can be increased to 185.2 MPa [26]. Other research results showed that the tensile strength and flexural strength of PLA structures reinforced with continuous carbon fibre are 245.4 MPa and 168.8 MPa, respectively [11]. The improvement in mechanical properties directly depends on the reinforcing material, its orientation in the structure, amount [27], the volume of the air voids [26], and the preparation of the carbon fibre before printing [24,28].

Currently, tensile and flexural properties are the most common research objects mentioned in the scientific articles [11,23,25], but other mechanical properties are equally important. Since AM is based on the layer-by-layer principle, the possibility to evaluate the interlayer adhesion becomes very important. Moreover, for specific composite structures, interlayer adhesion could be the main parameter which describes the strength of the structure. This is very important when the contact area between layers is decreasing. However, there is a lack of information related to the shear strength of 3D-printed composite structures. This research expands fundamental knowledge related to the shear strength of continuous carbon fibre-reinforced composite structures produced with additive manufacturing technologies. To determine adhesion between layers, various methods and joints could be used such as single lap joint (SLJ), double lap joint (DLJ), double butt lap joint, etc. The single-lap joint is the most common and widely used for the evaluation of shear strength. The performance of joints depends on various parameters such as overlap length, shear area, properties of materials, and surface preparation [29].

In this study, the shear strength of 3D-printed continuous carbon fibre-reinforced composites manufactured by fused deposition modelling technology was evaluated. The influence of shear area, overlap length, and production process parameters such as layer height and printing line width on shear strength were analysed. Finally, optical micrographs of the failure zones were analysed, and failure modes were determined. Research results can serve for the development of new optimised composite structures. Produced composite structures can be used in various fields in which they can successfully change plastic, short-fibre-reinforced, and even aluminium parts.

## 2. Materials and Methods

**Figure 1:**
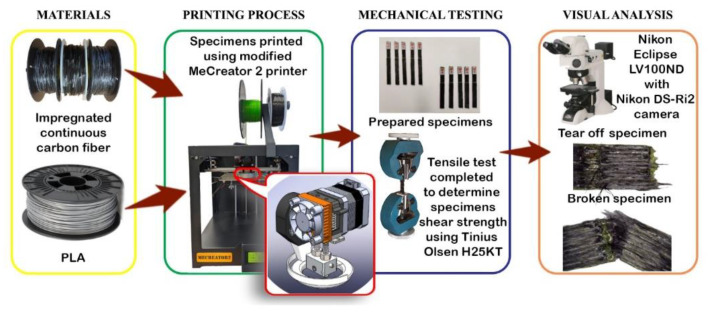
Research methodology.

The research consists of three stages: the printing process, mechanical testing, and visual analysis. The research scheme is presented in Figure 1. Continuous carbon fibre and thermoplastic filament were used for printing the samples. Before the printing process, the continuous carbon fibre was specifically prepared in the solution of methylene chloride and PLA thermoplastic. The material extrusion-based FDM technology was used for printing all samples. Finally, tensile tests and visual analysis were performed in order to determine shear strength, maximum shear force, and failure mode. Failure mode was determined according to ASTM D 5573 (Standard practice for classifying failure modes in fiber–reinforced–plastic (FRP) joints) standard, while shear strength was assessed according to recommendations of ASTM D 3163 (Standard test method for determining strength of adhesively bonded rigid plastic lap–shear joints in shear by tension loading) standard.

### 2.1. Materials

All composite samples were printed using PLA (D&R 3D Filament Ltd., Bristol, UK) filament with the diameter of 1.75 mm which was used as a matrix of composite material. Carbon fibre tow T300 B-1000 (Toray, Lacq, France) with one thousand filaments in the tow was selected as the reinforcing material. According to the manufacturer’s data sheets, the diameter of one filament is 7 µm, while the main mechanical properties of this fibre are as follows: tensile strength 3530 MPa, Young’s modulus 230 GPa, density 1.76 g/cm^3^, linear density 66 g/1000 m, strain at failure 1.5%. It is important to note that the carbon fibre tow was impregnated in a 10 percent solution of PLA and methylene chloride CH_2_ Cl_2_ (Eurochemicals, Kuprioniškės, Lithuania) before printing. The specific continuous carbon fibre (CCF) preparation procedure before printing improved the adhesion between matrix and reinforcing material and increased the mechanical properties of printed structures, also providing stability and reliability of the printing process. Impregnated carbon fibre tow was not prone to felting, it was stiffer, and more homogeneous (not prone to the distribution into separate filaments). This ensured more stable and reliable carbon fibre tow movement in the print head and its channels and reduced the chances of breakage or clogging during printing. In addition, the impregnated carbon fibre tended to adhere to the thermoplastic faster, easier, and more firmly during the printing process. Scientific research confirmed the existence of the correlation between the concentration of the solution used for the impregnation and the mechanical properties of printed composite structures, i.e., the higher concentration of the impregnation solution, the better were the mechanical properties [28]. The quality of the impregnation process depends on several basic parameters: inlet and outlet nozzle diameters, the constancy of pulling speed, and drying temperature. The carbon fibre tow used in this research was prepared using the impregnation process with the following parameters: pulling speed 30 rpm, drying temperature 220 °C, inlet and outlet channel diameter 0.6 mm.

### 2.2. The Printing Process of Composite Samples

**Figure 2:**
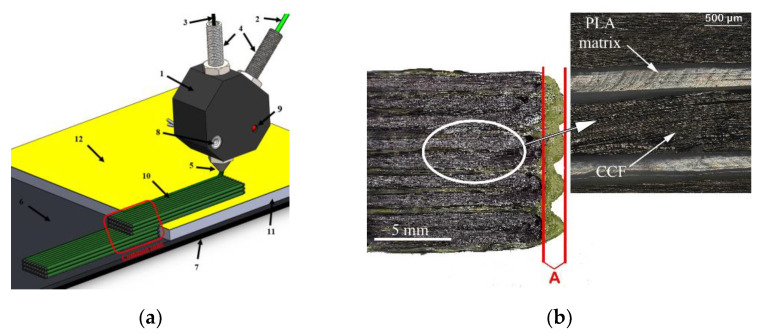
(**a**) Printing scheme and (**b**) displacement effect.

FDM technology printer MeCreator 2 (Geeetech, Shenzhen, China) was chosen to print the composite samples. The scheme of the printing process is shown in Figure 2a. It should be mentioned that the device initially was adapted to print single thermoplastic material only; thus, it needed a modification to print composite materials. Therefore, designed and manufactured a completely new heating element (1), and a modified cold zone of the print head was used during experiments. During the printing process, the thermoplastic (2) and the continuous carbon fibre tow (3) were inserted into the heating element simultaneously through two separate inlet channels (4). The matrix and the reinforcing material were mixed in the heating element just before the composite filament was extruded through the printing nozzle (5) onto a boron silicate glass plate (6) which was placed on a metal printing platform (7). It should be noted that matrix material (PLA) was fed to the print head using a standard filament feeding system.

The carbon fibre tow was fed due to the movement of the print head and the interlayer adhesion to the printing platform. A constant temperature in the printing head was maintained by an electric heating element (8) and controlled by a thermocouple (9). In order to increase the interlayer adhesion of the composite line to the printing platform and to reduce the displacement effect (Figure 2b), the glass surface was coated with a thin layer of 3DLAC (Laboratorios 3D Print, San Cristobal de Entrevinas, Spain) adhesive spray before printing. The displacement effect occurred because impregnated carbon fibre tow was slightly pulled when the print head changed its direction. The displacement resulted in the reduced sample length. Value A indicates the change in length that occurred during the printing process. This effect cannot be completely eliminated, but it can be reduced by increasing the cooling of this zone especially during changes of direction, by reducing the printing speed, or using additional materials that increase the interlayer adhesion between the platform and the printed structure. In this research, additional adhesive layer between the printing bed, the first printed layer, and adjusted printing parameters (printing speed and cooling) were used in order to minimise the effect of displacement. It worth mentioning that printing speed and cooling rate were changed experimentally, for example, printing tests were made with printing speed changing from 1 mm/min to 5 mm/min and cooling rate from 50% to 100%. The final production parameters were selected according to printing quality, efficiency, and displacement results. As mentioned earlier, at the beginning of the printing process, plastic was extruded and adhered to the printing platform, together with the carbon fibre tow. As the print head moved in the direction of the *x*–*y* axis, the composite line repeated the path of the printing head and formed the object. However, during changing the direction of movement, especially when the direction was changed by more than 120°, displacement effect appeared in the sample.

In order to identify the shear strength, single lap-joint samples (10) were chosen for printing. Samples of this type were chosen due to their simplicity and popularity for the purpose of the identification of the shear strength in composite structures. In order to print such samples on a modified printer, a glass plate (11) of an appropriate thickness (depending on the height of the layer of the sample to be printed) was added to the platform at the end of the first part during the printing process. A Kapton tape (12) was glued to the surface of the plate to increase the interlayer adhesion between the printed sample and the platform. The Kapton tape was also covered with a thin layer of 3D LAC adhesive before printing. All samples used in the research were printed on the same printer, using the same materials and constant printing parameters. However, layer height and width of the printing line were changed in order to evaluate their influence on shear strength. The main production process parameters are presented in Table 1.

**Figure 3:**
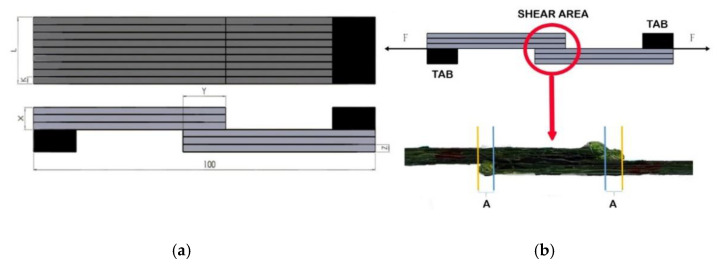
Schematic view (**a**) of the specimen and (**b**) of the tensile test and shear area.

In order to perform a statistical evaluation of the results, it was decided to print five samples in each group (Figure 3a), keeping a constant sample length of 100 mm and the three printed layers in each part of a single lap joint. However, it was decided to change four other parameters: layer height Z (from 0.3 to 0.4 mm), the width of printing line K (from 1 to 1.2 mm), the overlap length Y (from 10 to 20 mm, increasing the length in 5 mm increments), and the number of printed lines in the width of the specimen L (9 and 11 lines). A total of 120 samples were printed using the abovementioned parameters. A summary of the printed samples is provided in Table 2 and Table 3. In Figure 3b, the tension test scheme and 3D model of the specimen are presented. Distance A indicates the change in length that occurred during the printing process. In this area, a small amount of matrix material residues could be observed, but carbon fibre was not embedded here. It is worth mentioning that such residues of matrix material were detected in all groups of samples, but their influence on mechanical properties was insignificant. Therefore, distance A was not included in the measurement of overlap length.

All printed samples were grouped according to the number of lines in their width. The number of printed lines (width of samples), as well as the overlap length, had a direct influence on the shear area and thus on the shear force. Each group of samples consisted of 12 subgroups with different layer heights, widths of the printing line, and overlap lengths. Table 2 shows all sample subgroups with nine printing lines in width.

Table 3 shows subgroups of samples with 11 printing lines in width. As it can be seen from this information, the actual shear area increased by 30% on average, having increased the number of printing lines from 9 to 11. The largest actual increase of the shear area was about 41% and was observed at specimens with the nominal overlap length of 10 mm.

A larger increase of the shear area in these groups of samples was observed as a result of the displacement effect of the printing line that occurred during printing. During the printing process, the sample length and the shear area length decreased by an average of 5 mm as the print head changed its direction. This means that the sample shortened by 2.5 mm on one side. Aiming to print a 100 mm long sample, the CAD model was designed 5 mm larger in length, i.e., 105 mm. The shear area of each sample was measured separately. It was measured by making the assumption that the shear area had a rectangular shape. Finally, the actual overlap length and width of the shear area were measured using a measurement microscope TM-505 (Mitutoyo, Kawasaki, Japan). Mean overlap lengths for different groups of samples are presented in Table 4.

The overlap length of the shear area for the adhesion test was chosen experimentally. Samples with a nominal overlap length of less than 10 mm were very difficult to print, while samples with an overlap length of more than 20 mm were difficult to separate because they broke before separation. Therefore, it was decided that the overlap length of the shear area would be in the range of 10–20 mm when determining the shear strength.

### 2.3. Test Methodology

The tension test was performed on an H25 KT (Tinius Olsen Ltd., Redhill, UK) universal testing machine, with mechanical clamps, at the speed of 2 mm/min. During the experiment, the machine was equipped with a 1 kN load cell model (HTE-1000 N). Tinius Olsen Ltd.’s Horizon software was used for machine control, data acquisition, and analysis. During the experiments, print head displacement, force, and time were evaluated. The shear strength value was calculated after the tension test by using the formula presented below:

τ = F/A(1)

where F—maximum shear force, N, and A—shear area, mm^2^. It was decided to use tabs glued at the ends of the printed samples. The use of tabs helped to reduce the effects of cutting, bending, and torsional forces during the test. The scheme of the prepared sample and added tension forces is shown in Figure 3b. Dimensions of the tabs were as follows: thickness 0.9 or 1.2 mm depending on the sample layer height, length 15 mm regardless of the sample parameters, and width equal to the sample width. Tabs were printed with an FDM machine from PLA thermoplastic and glued to the specimen using ethyl 2 cyanoacrylate universal glue, having previously removed all plastic deposits with sandpaper and preparing the contact surface of the sample for better adhesion between the tab and the sample. After the shear strength test, a visual analysis of the samples was performed to determine the failure modes. An optical microscope Eclipse LV100 ND (Nikon, Tokyo, Japan) with a DS-Ri2 (Nikon, Tokyo, Japan) digital camera and a wide-field objective was used for visual analysis.

## 3. Results and Discussion

### 3.1. Interlayer Adhesion

**Figure 4:**
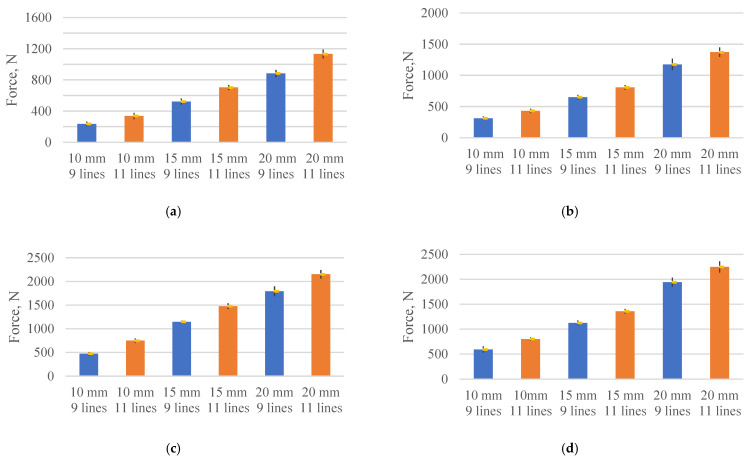
Diagrams of the shear force for the specimen with different line numbers and overlap lengths: (**a**) 1.2–0.4; (**b**) 1–0.4; (**c**) 1.2–0.3; (**d**) 1–0.3.

During the experiment, all samples were successfully separated. None of the samples was broken above or below the shear area. This means that the average tensile strength of the test structure was not exceeded during the test; the average tensile strength of the composite material was determined experimentally and accounted for approximately 204 MPa. The test resulted in finding out the force necessary for the separation of the specimen. Detailed results are presented in Figure 4, having them grouped according to the printing line width and the layer height. Results of the samples with 9 lines per width are presented in blue colour, while results of samples with 11 lines are presented in orange colour. As can be seen from the diagrams in Figure 4, the main factors having an influence on the value of the shear force are the overlap length and number of lines. Obviously, these two parameters also directly affect the shear area. Assessing how the results change when the sample layer height decreases from 0.4 mm to 0.3 mm, it can be seen that the amount of force required for separation increases significantly. The average shear force is 235.6 N for samples printed using 1.2 mm–0.4 mm (line width-printing layer height) parameters, nine printing lines, and nominal overlap length of 10 mm (Figure 4a); however, when the layer height changes to 0.3 mm, the shear force increases to 470.33 N (Figure 4c). The change of the layer height from 0.4 mm to 0.3 mm allows increasing the shear force about two times. A similar upward tendency is observed in the sample groups printed with the different layer heights and with a constant line width of 1 mm. For example, the shear force increases from 314 N to 593.3 N or 88 percent for samples with nine lines and 10 mm overlap length when the layer height changes from 0.4 to 0.3 mm. This upward tendency does not depend on the shear area, the number of printing lines, or the overlap length, and remains approximately the same in all sample groups, as seen in Figure 4.

Moreover, from the presented results, it can be stated that printing layer height is a very important parameter which has a significant influence on the shear force of composite structure. Another important printing parameter that affects the shear force in composite products is the width of the printing line. The change of the line width from 1.2 mm to 1 mm results in an increase in the shear force, approximately by 30%. Comparing the results of samples with 11 printing lines and the overlap length of 20 mm, it is obvious that the value of force required for separation increases from 1134 N to 1373 N, or 21 percent, when the layer height is 0.4 mm, and the line width decreases from 1.2 mm to 1 mm. A similar trend can be seen with samples when the line width changes from 1.2 mm to 1 mm, but layer height is 0.3 mm. Assessing the influence of shear area on the shear force, it can be seen that shear force increases from 235.6 N to 1133.6 N when the shear area changes from 38.5 mm^2^ to 175.7 mm^2^, respectively. This behaviour is observed for samples with 1.2 mm line width and 0.4 mm layer height. On the other hand, when increasing the shear area by 4.6 times, the shear force proportionally increases by 4.8 times. In the group of samples with 1–0.4 (line width-printing height) parameters, the force changes from 314 N to 1373 N by increasing the shear area from 37.7 mm^2^ to 151.8 mm^2^. In this case, the force required for the separation increases 4.4 times, having increased the shear area by 4.1 times. A similar tendency is observed in samples with a line width of 1.2 mm and the layer height of 0.3 mm, where the shear force increases from 470.3 N to 2150.6 N when the area increases from 38.5 mm^2^ to 175.7 mm^2^. As is seen from the presented results, when the shear area increases 4.6 times, the shear force increases proportionally 4.6 times. The same tendency is observed in samples with a line width of 1 mm and a layer height of 0.3 mm. When increasing the area from 37.7 mm^2^ to 151.8 mm^2^, the force increases from 593.3 N to 2247.3 N. Thus, when the shear area increases 4 times, the force required for separation increased 3.8 times. It can be concluded that the increase in the shear area results in a proportional increase in the force required for separation. The observed linear dependency of the shear force in the shear area allows predicting the properties of 3D-printed composite structures of this type. It can be concluded that from the two printing parameters, such as the printing line width and the layer height, the latter has a bigger influence on the value of the shear force. It can be assumed that by reducing the layer height and the width of the printing line, the amount of air voids in the sample decreases. This assumption is based on a widely described and discussed plastic behaviour while printing by the FDM method [30,31,32]. For a comprehensive analysis of the influence of air voids on mechanical parameters in composite structures, computed tomography or ultrasound testing should be performed. Assessing the results, it becomes clear that a decrease in these printing parameters allows a significant increase in the interlayer adhesion. These studies are very important since they allow predicting the behaviour of 3D-printed composite structures with various shear areas. It can be concluded that the value of the shear force increases linearly when the shear area increases. Increasing the number of lines from 9 to 11, the value of the shear force increases by an average of 179.6 N in the group of printed samples with the printing line width of 1.2 mm and the layer height of 0.4 mm, whilst in the group of samples with the line width of 1 mm, it increases to 211.8 N. When the layer height is reduced to 0.3 mm in these groups of samples, the average increase in shear force is 336.0 N, and 330.8 N, when the line width varies from 1.2 mm to 1 mm. The same tendency is observed when increasing the overlap length. In the samples printed with 1.2–0.4 (line width-printing height) parameters, the value of the shear force increases by an average of 360.9 N by increasing the overlap length from 10 to 20 mm, while in the samples printed with 1–0.4 parameters, the increase is 451.2 N. In the samples with the layer height of 0.3 mm, the increase of the force required for separation is almost the same, i.e., 681.4 N and 699.2 N, when the printing line width varies from 1.2 mm to 1 mm. These results also show that the lower layer height provides stability and reliability to the printing process, as well as to the printed structure and its parameters, as evidenced by the lower distribution of the data obtained. Accordingly, printing with a smaller layer height allows for better interlayer adhesion and stability between the individual lines. Therefore, aiming to print stronger, more resilient, and reliable composite structures, the height of the printing layer must be chosen as low as possible after the appropriate consideration of the technological possibilities of the process.

**Figure 5:**
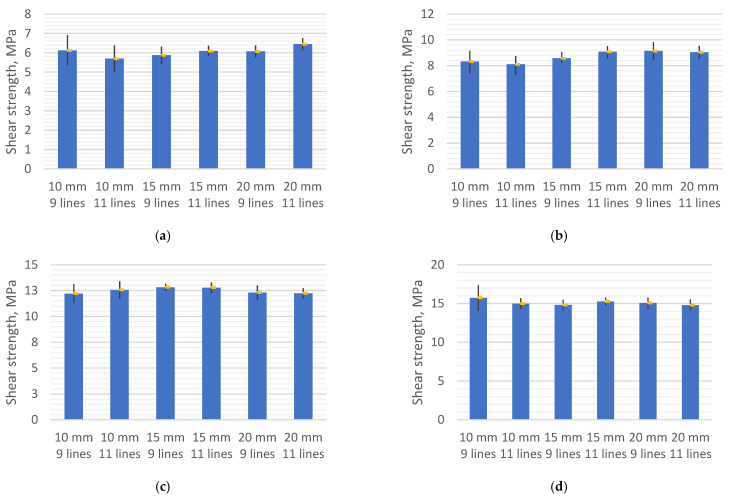
Diagrams of the shear strength for the specimen with different line numbers and overlap lengths: (**a**) 1.2–0.4; (**b**) 1–0.4; (**c**) 1.2–0.3; (**d**) 1–0.3.

As can be seen from the diagrams in Figure 5, the shear strength is almost independent of the shear area. Samples printed with the same printing parameters show slight changes in shear strength results. This behaviour of 3D-printed composite material is interesting and could be explained with earlier studied dependency between shear force and shear area. However, this mechanical property is greatly influenced by the layer height and the printing line width. For example, samples printed with a layer height of 0.4 mm and a line width of 1.2 mm have an average shear strength of 6.1 MPa, while reducing the line width to 1 mm allows increasing shear strength to 8.7 MPa, which constitutes a significant increase of 42.6%. Assessing the effect of the line width in samples printed with the layer height of 0.3 mm, it has been noticed that the shear strength increases from 12.5 MPa to 15.1 MPa, i.e., about 21%, when the line width varies from 1.2 mm to 1 mm. The change in layer height has an even greater impact on the shear strength of 3D-printed composite structures. When layer height is reduced from 0.4 mm to 0.3 mm, shear strength increases, on average, about two times, for example, from 6.0 MPa to 12.5 MPa and from 8.7 MPa to 15.1 MPa, when the line width is 1 mm and 1.2 mm, respectively. The distribution of results is one more important factor which needs to be considered. As can be seen from the diagrams in Figure 5, the largest variation of data is observed when the overlap length is 10 mm, regardless of the layer height and the line width. This is because the actual reduction in the length of the shear area, occurring due to the displacement of the printing line, is larger and more variable than in larger overlap lengths. Larger variation and shortening happens due to the shorter printing path and the reduced cooling time of the printed area (loop). Therefore, when designing 3D-printed composite structures which are produced using the additive manufacturing technology mentioned in the article, it is necessary to avoid a small overlap length. After the analysis of results, it can be concluded that the shear strength of 3D-printed composite structures is highly affected by process parameters such as printing line width and layer height.

### 3.2. Visual Analysis

**Figure 6:**
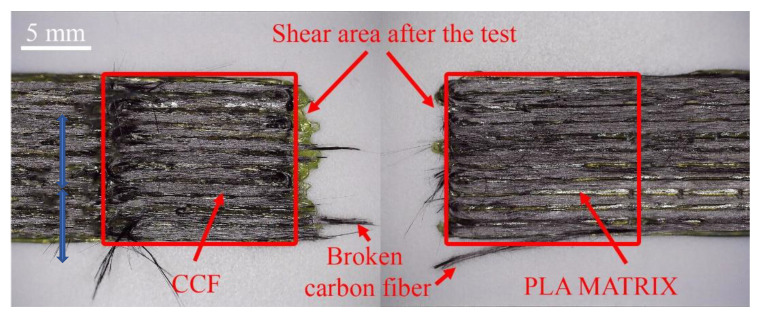
The 11 printing lines of the G3 group specimen after the test.

After determining the shear strength of the samples, a visual analysis of the separated samples was performed. A Nikon Eclipse LV100 ND optical microscope with a Nikon DS-Ri2 digital camera and a wide-field objective was used for analysis. When determining the shear force, it was observed that the samples with overlap length of the shear area of up to 20 mm only, regardless of the printing parameters, tend to separate. Figure 6 shows an image of a sample belonging to the G3 group of 11 lines after the shear test. Similar to the presented image in Figure 6, most of the specimens separated smoothly without causing fractures, visible cracks, large tearing of the carbon fibre from the matrix, or other visually visible structural changes. This tendency is observed in all groups of samples.

**Figure 7:**
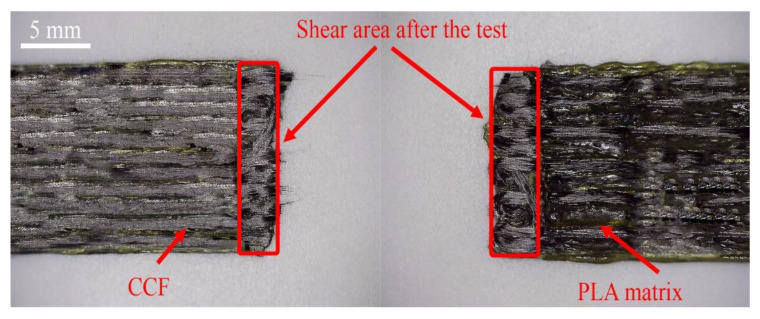
The 11 printing lines of the G4 group specimen after the testing.

This suggests the presumption that the sample is affected only by shear forces during the test. However, assessing the interlayer adhesion, it can be seen that lines of plastic and impregnated carbon fibre are visible on the surface of the layers. Obviously, better mutual adhesion can be achieved due to a higher amount of matrix material in the shear area, a lower layer height, or a printing line width. Using lower printing parameters, i.e., at a lower layer height and line width, the structural integrity of the structure is better because the carbon fibre is embedded more firmly in the thermoplastic matrix during the manufacturing process. Accordingly, Figure 7 shows the picture of the sample with a small shear area. Although no visual damage is visible, the small overlap length is reduced even more due to the displacement effect mentioned above, and it does not allow achieving better adhesion results.

**Figure 8:**
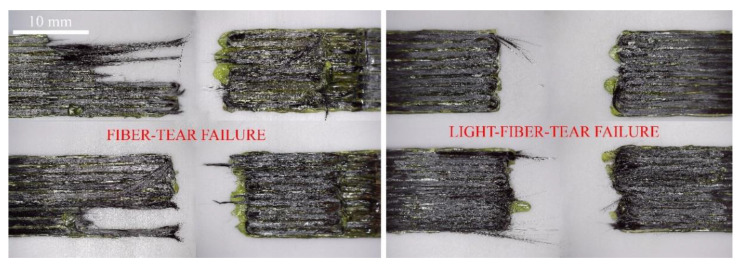
Observed failure modes.

However, some specimens with an overlap length of 20 mm have a tendency to separate with different failure modes. Therefore, it was decided to analyse failure modes of samples after the shear test according to the ASTM D 5573 standard. The results indicate that most of the samples show light-fibre-tear failure modes. Examples of these failure modes are presented in Figure 8. Fibre-tear failure occurs exclusively within the fibre-reinforced polymer matrix, and it is characterised by the appearance of reinforcing fibres on both separated surfaces. Light-fibre-tear failure occurs within the fibre-reinforced polymer substrate near the surface and is characterised by a thin layer of the FRP resin matrix visible on one side of the specimen, with few or no carbon fibres transferred from one part to another. However, after analysis, a clear tendency is not observed, i.e., in several groups of samples two different failure modes are found. These groups of samples were produced with different printing parameters when the overlap length was 20 mm. In such groups of samples, light-fibre-tear and fibre-tear failure modes are observed. Despite the fact that clear tendency is not identified, it should be mentioned that most of the samples printed with 0.3 mm layer height and 1 mm line width show fibre-tear failure modes. This behaviour can be explained by improved adhesion between layers which is observed during shear force analysis.

**Figure 9:**
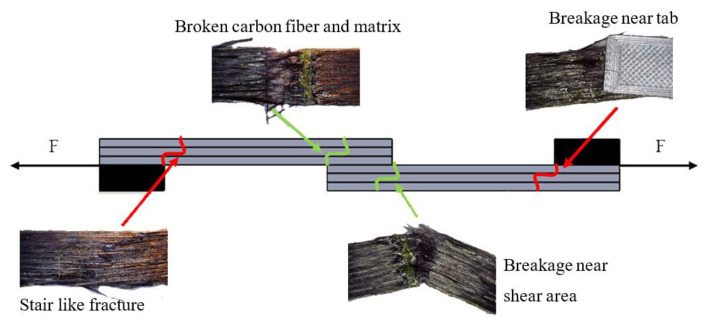
Schematic diagram of specimen fracture patterns.

Samples with an overlap length above 20 mm were not included in the research because they tended to break before they could be separated during the test. In this case, two different types of breakages are observed: breakage at one of the tabs and breakage at the ends of the shear area, which can be seen in Figure 9. If the breakage at the tabs can be explained by the resulting stresses in the compression zone, then the breakage at the contact zone clearly indicates that the tensile strength of the sample is exceeded.

No significant effect of printing parameters on the breakage of the samples and the nature of the separation is observed. Assessing the test results of the samples, it can be stated that the shear force of the samples exceeds the maximum tensile strength, but it is less than the force required for separation. Aiming to examine samples with a larger shear area and to determine if they have similar tendencies, thicker samples should be printed.

## 4. Conclusions

The force required to separate 3D-printed composite structures, reinforced using continuous carbon fibre, which directly depends on the layer height, width of the printing line, and shear area, was experimentally identified during the research. Depending on these variable parameters, it ranges from 235 N to 1133 N in samples with the printing line width of 1.2 mm and the layer height of 0.4 mm, and from 314 N to 1373 N in samples with the line width of 1 mm and the layer height of 0.4 mm, whereas in samples with the layer height of 0.3 mm, the shear force varies from 470 N to 2150 N, and from 593 N to 2247 N, when the line width varies from 1.2 mm to 1 mm. Having reduced the layer height from 0.4 mm to 0.3 mm, the value of force required for separation increases two times on average. Having reduced the line width from 1.2 mm to 1 mm, the force increases by 30% on average. When the shear area increases, the value of shear force increases proportionally. Increasing the number of lines in the sample from 9 to 11, the value of shear force increases by 179.6 N, and 211.87 N, on average, when the layer height is constant at 0.4 mm and the line width varies from 1.2 mm to 1 mm. Whilst reducing the layer height to 0.3 mm in the group of samples, the average increase in shear force is 336.07 N, and 330.80 N, when the line width varies from 1.2 mm to 1 mm. The shear strength was also calculated during the research, which depends on the printing parameters only but is almost independent of the shear area. The shear strength averages 6.1 MPa in samples printed with the line width of 1.2 mm and the layer height of 0.4 mm, but having reduced line width to 1 mm, the shear strength increases to 8.7 MPa. The shear strength of sample groups printed using the layer height of 0.3 mm is 12.5 MPa and 15.1 MPa, respectively, when the line width varies between 1.2 mm and 1 mm. Visual analysis of the samples reveals that all samples, regardless of the printing parameters or the length of the shear area, separate smoothly without fractures, cracks, tearing of the carbon fibre from the matrix, or other visually visible structural changes. This tendency prevailed in all groups of samples.

The results show that lower layer height provides stability and reliability to 3D-printed composite structures and their mechanical properties, ensuring better interlayer adhesion between the matrix and the reinforcing material. Thus, aiming to print stronger, more resilient, and reliable composite structures, the height of the printing layer should be chosen as low as possible. Moreover, the results obtained can be used for the design and modelling of complex composite 3D-printed structures.

## Figures and Tables

**Table 1:** Printing parameters.

Printing Settings	Value, Description
Nozzle diameter	1.5 mm, stainless steel
Adhesive layer	3DLAC spray
Fan use (cooling)	80%
Extruder temperature	210 °C
Extrusion multiplier	0.7
Build platform temperature	80 °C
Printing speed	180 mm/min
First layer printing speed	144 mm/min
Print orientation	Flat
Fiber orientation	Unidirectional 0°
Infill ratio	100%
Layer height	0.3 mm and 0.4 mm
Width of the printing line	1 mm and 1.2 mm

**Table 2:** Subgroups of the printed specimens with nine lines.

Subgroup	G1	G2	G3	G4	G5	G6	G7	G8	G9	G10	G11	G12
Layer height, mm	0.3	0.3	0.3	0.4	0.4	0.4	0.3	0.3	0.3	0.4	0.4	0.4
Width of the printing line, mm	1	1	1	1	1	1	1.2	1.2	1.2	1.2	1.2	1.2
Overlap length, mm	10	15	20	10	15	20	10	15	20	10	15	20
Shear area, mm^2^	37.7	75.9	128.8	37.7	76.2	128.9	38.5	89.2	145.6	38.56	89.3	145.6

**Table 3:** Subgroups of the printed specimens with 11 lines.

Subgroup	G1	G2	G3	G4	G5	G6	G7	G8	G9	G10	G11	G12
Layer height, mm	0.3	0.3	0.3	0.4	0.4	0.4	0.3	0.3	0.3	0.4	0.4	0.4
Width of the printing line, mm	1	1	1	1	1	1	1.2	1.2	1.2	1.2	1.2	1.2
Overlap length, mm	10	15	20	10	15	20	10	15	20	10	15	20
Shear area mm^2^	53.3	89.0	151.8	53.4	88.9	151.9	59.4	115.5	175.7	59.5	115.6	175.6

**Table 4:** Difference of the shear area length.

Nominal Overlap Length, mm	Real Overlap Length, mm	Difference between Nominal and Real Overlap Length, %
10	4.8	52
15	9.7	35
20	15.1	24.5

## Data Availability

The data presented in this study are available on request from the corresponding author.
